# Digital Workflow for Producing Hybrid Posts and Cores

**DOI:** 10.3390/healthcare11050727

**Published:** 2023-03-02

**Authors:** Paula Perlea, Cosmin Stefanescu, Omar-Andrei Al-Aloul, Cezar Ionita, Alexandru-Eugen Petre

**Affiliations:** 1 Department of Endodontics, Carol Davila University of Medicine and Pharmacy, 050474 Bucharest, Romania; 2 Department of Prosthodontics, Carol Davila University of Medicine and Pharmacy, 050474 Bucharest, Romania

**Keywords:** CAD-CAM, digital workflow, fiber post, post and core, zirconia

## Abstract

A novel and straightforward digital workflow is described to aid clinicians in producing in-office hybrid posts and cores. The method is based on scanning and using the basic module of a computer-assisted design and computer-assisted manufacturing (CAD-CAM) software program for dental applications. The applicability of the technique in a digital workflow is the simplicity of in-office production of a hybrid post and core that can be delivered to the patient in the same day.

## 1. Introduction

A suitable method is necessary to restore the tooth after endodontic treatment, provide a coronal seal, and protect the residual dental structure. Posts and cores are used to provide retention and support for the crowns when endodontically treated teeth are severely damaged. Teeth with two or more compromised walls need to be restored with posts to increase the retention and stability of the final crown [1,2].

Computer-aided design and computer-aided manufacture (CAD-CAM) technologies are advancing rapidly in all fields of dentistry, and help doctors to reduce work time, increase precision, and accelerate production processes. A challenge for digital workflows is to obtain a CAD-CAM post and core without using conventional impression material. Generally, customized posts and cores are obtained by scanning plaster models [3,4]. Some studies have suggested a different digital workflow in which the technician directly scans the silicon impression taken by the doctor [5]. Digital workflows for producing posts and cores provide the advantages of simplicity, reduced storage, easy transfer of data, patient comfort, and a reduction in chairside time and costs. Additionally, digital workflows eliminate all the steps required for manipulation of the impression before and after taking it.

Zirconia and fiber posts are indicated for anterior teeth to avoid esthetic failures. Fiber posts have superior biomechanical properties in comparison with metal or zirconia posts and cores. This is because of their physical properties, which are comparable to natural dentine and allow a better distribution of occlusal forces [6]. Additionally, the risk of vertical root fracture is reduced in comparison with metal or full ceramic posts and cores [7,8]. In contrast, zirconia can be used for producing customized posts and cores that fit into irregular post spaces [3]. Both zirconia and fiber posts can be bonded with MDP-based resin cements. Many studies showed that resin cements containing MDP can bond strongly with sandblasted zirconia and also with fiber posts [1,9]. Bonding together zirconia and fiber posts with MDP-based resin cements has also been described by other authors [4,10]. The chemical reaction is confirmed by the presence of the hydrophobic 10-carbon spacer chain of 10-MDP which coats the zirconia surface when the primer is applied [11]. In contrast with zirconia, which does not react at silanation, fiber posts can be used with a silane coupling agent. They form chemical bonds at the interface with resin cement. Hydrolysis of the methoxy groups form covalent siloxane bonds (-Si-O-Si) with the post [12].

This article describes a novel and simplified method for in-office scanning of the post and core preparation, without using a conventional impression material. A step-by-step workflow will be described by using Exocad (Exocad GmbH, Darmstadt, Germany), one of the most used dental CAD software applications.

## 2. Technique

**Figure 1:**
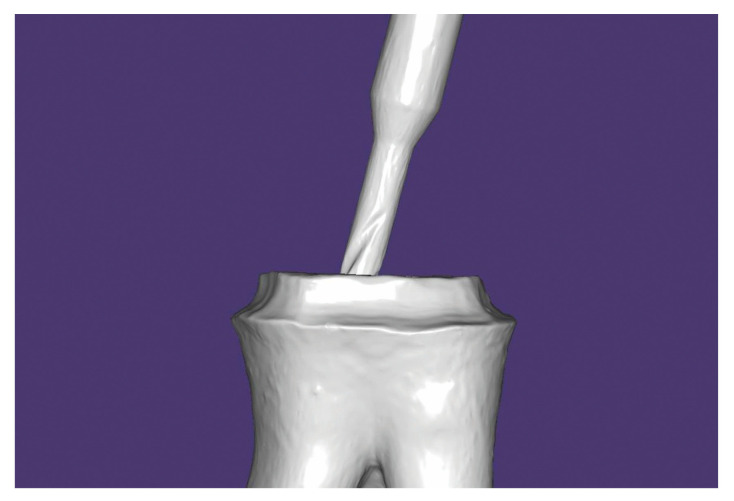
Scan of the tooth with the bur placed into the canal.

**Figure 2:**
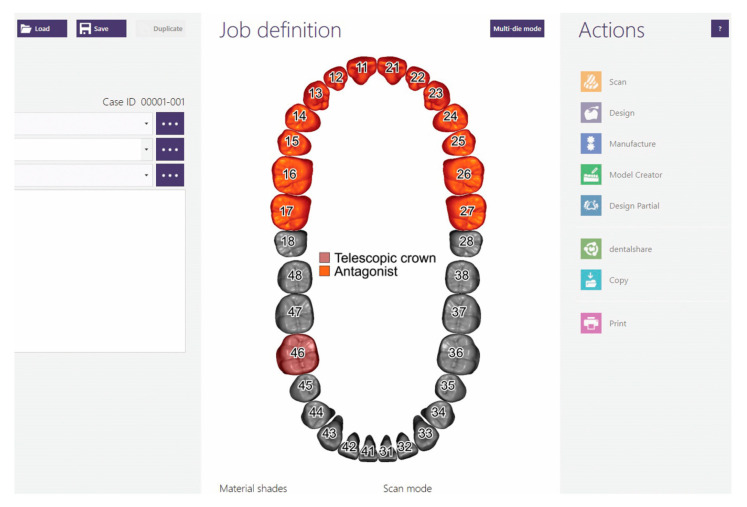
Job definition in CAD software.

1. Finish the endodontic treatment at microscope. Perform the preparation of the canal for the post and core with a standardized bur, part of a fiber post kit. Additionally, prepare the coronal part of the tooth according to the guidelines for post and core preparation [13]. Finish, then check and clean the preparation.

2. Take a digital impression using the intraoral scanner TRIOS 3 (3Shape, Copenhagen, Denmark). Create the patient scanning chart, select “New case” and “In-house Lab” for the default laboratory connection. In the software’s patient chart, select the tooth and click Anatomy > Crown to enable the software to scan it in HD mode for higher precision of the canal preparation. Additionally, enable the “Pre-preparation” button from the right side of the screen. Now the software can scan the fourth STL entitled “Lower Pre-Preparation”.

3. Begin by scanning the antagonists and then scan the tooth with the bur placed into the canal as a “Lower Pre-Preparation” (Figure 1) using the intraoral scanner. The span of the digital impression can be at minimum 2–3 adjacent teeth mesial and distal. Next, the software prompts you to mark the tooth, and a blue overlay will appear over the prepared tooth. Now the calibrated bur is taken out of the canal and the tooth is scanned. The blue area from the previous scan is removed so there is no need to scan the whole quadrant again. Scan the occlusion as usual. The software will post-process four scans: “Lower”, “Lower Pre-Preparation”, “Upper”, and “Occlusion”.

4. From the patient’s chart, right click on the scan and select “Export” > “Scans”. Then, create a new folder for the files. For better organization, create a sub-folder for each patient, change the file type from Digital Imaging and Communications in Medicine (DCM) to Standard Triangle Language (STL), name the files according to the folder name and click “Save”. The files will be saved as LowerJawScan.stl, LowerPrePreparationScan.stl, UpperJawScan.stl, and BiteScan.stl.

5. A computer-aided design software (Exocad, Darmstadt, Germany) is used to design the hybrid post and core. Select a suitable client and technician and give a name to the Job Definition. The name can be the same as the folder created previously. The prepared tooth is declared as Telescopic Crown, the opposing arch is declared as Antagonist, and the scan mode is declared as Digital Impression scan (Figure 2).

**Figure 3:**
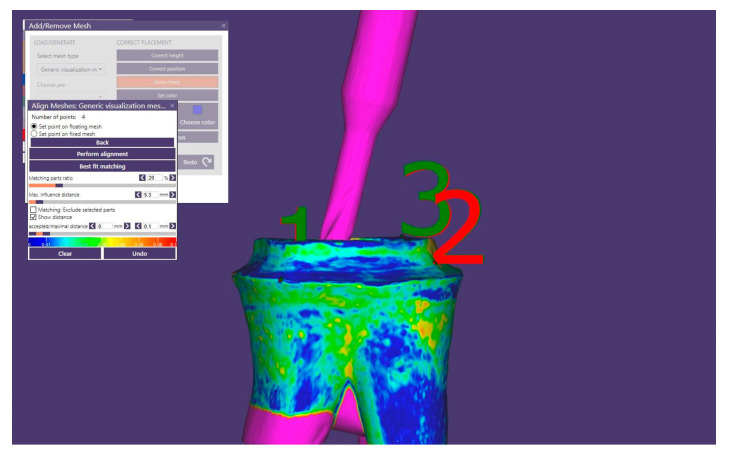
Overlapping scans in CAD software.

Click “Save and Design”. When the CAD window opens, import LowerJawScan.stl as Lower jaw, and UpperJowScan.stl as Upper jaw. Set the scan data orientation and click on Expert mode, where the Add/Remove mesh option can be accessed. Scan named LowerPrePreparationScan.stl is imported as “Generic visualization mesh” and overlaps with the scan without a bur. Both scans will now be in the same position (Figure 3).

**Figure 4:**
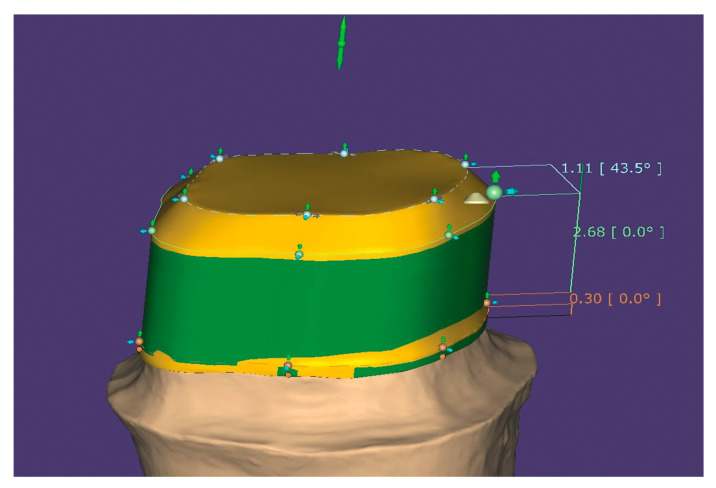
Design the core in CAD software.

**Figure 5:**
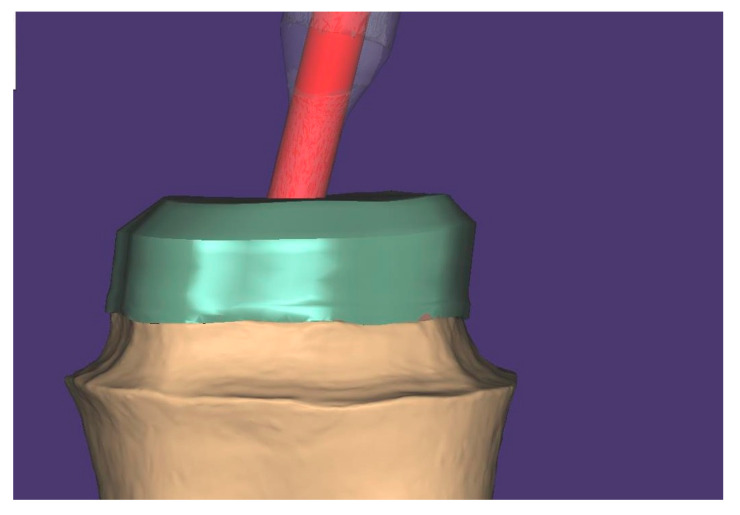
Digitally drill the core according to the orientation of the fiber post.

**Figure 6:**
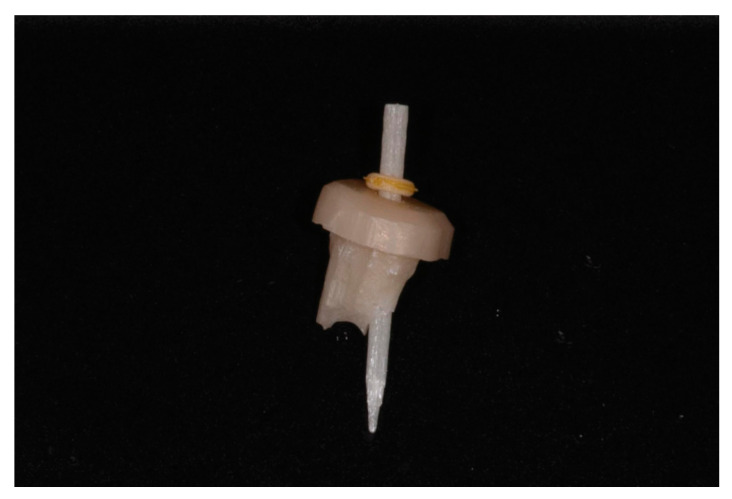
Check zirconia core and fiber post.

**Figure 7:**
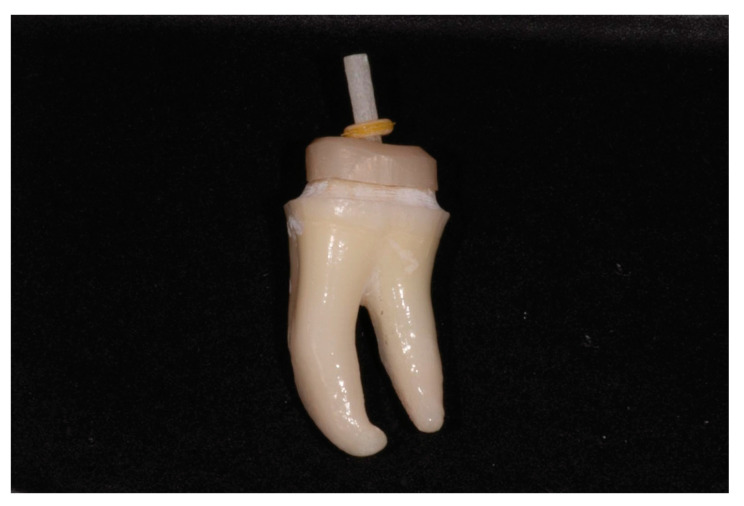
Zirconia core and fiber post simultaneously seated in the preparation.

6. Exit the Expert module and proceed further with the design of the core: margin line detection and bottoms. A cement gap of 0.08 mm is recommended between the core and the walls of the preparation. The CAD software will automatically generate a tooth model from the library that can be moved, scaled, and rotated into the correct position. This crown is edited with “Free Forming”, adapted in occlusion and approximal. In the next step, the software will generate a “Primary telescope” in accordance with the morphology of the future crown and allow the operator to set the insertion axis. The core geometry will be designed according to this axis. Adjustments of the final shape can be performed in the “Free Forming Telescope” step (Figure 4). The telescope is merged and saved.

7. The core generated by the software has to be drilled according to the post orientation. For this procedure, the mesh with the post will be activated and its translucency will be reduced by 75%.

8. Access “Free form reconstructions” and select “Attachments > Remove > Parametric design (extrusion) > Circular”. The radius of the cylinder will be set according to the diameter of the fiber post plus 15 microns to allow space for cement. The orientation will be set by clicking on “Set from view” with the button “Allow any changes” activated. A hole through the core is created, according to the orientation and diameter of the fiber post (Figure 5).

9. The CAD software automatically exports an STL file to the designated folder. Send the STL file to a four-axis milling machine (CEREC MC XL, Dentsply Sirona, Germany), mill a zirconia block (Katana Zirconia Block, Kuraray Noritake, Japan), and sinter the milled zirconia core in a high-speed furnace (CEREC SpeedFire, Dentsply Sirona, Germany).

10. First, evaluate the fit of both pieces (Figure 6). The fiber post should slide passively through the zirconia core. Second, sit the zirconia core separately in the prepared tooth, respecting the core’s insertion axis. The zirconia piece should sit passively due to the CAD software’s capacity to block out undercuts. This step should not require adjustments. Once the zirconia core is seated, place the fiber post through the core’s canal. The post should slide along its dedicated canal in the zirconia core (Figure 7). Then, simultaneously cement them with a dual-cure resin cement compatible with both zirconia and fiber posts, such as Panavia V5 (Kuraray Noritake, Tokyo, Japan). Apply resin on the inner surface of the zirconia core, through the canal, and on the outer surface of the post. Push the fiber post through the canal after the zirconia core is placed into the preparation and polymerize the resin. Use an MDP primer agent as recommended (Clearfil Ceramic Primer, Kuraray Noritake, Japan). After the cement sets, cut the fiber post at the level of the core with a high-speed bur.

## 3. Discussion

This method represents a quick and straightforward way to precisely design a hybrid post and core drilled with the exact angulation of the canal. This angulation is subject to an error of 5–10 microns, depending on the overlapping of the two maxillary scans. The error is predictable and does not have any major impact on the definitive restoration [14].

The method described is a novel tool to overcome the shortcomings of direct scanning procedures. Studies showed that errors frequently occur when the scanner records narrow and deep surfaces. Is difficult to reach with the white light of the scanner into the deepest areas of the post space [15,16].

**Figure 8:**
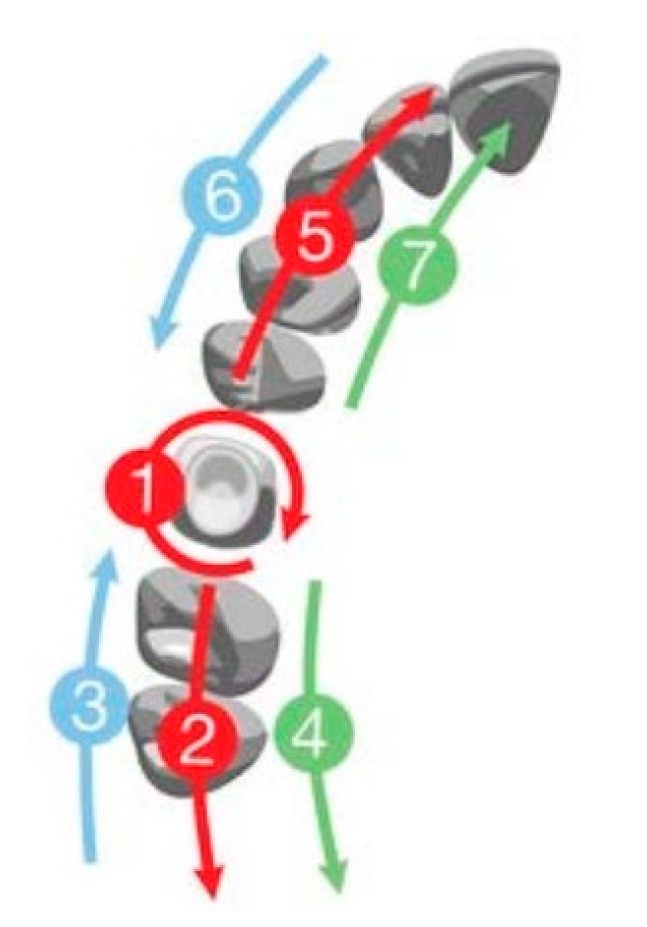
Scanning strategy.

The accuracy of the method depends on the correct scanning of the bur placed into the canal. Clinicians can use a TRIOS 3 (3Shape, Copenhagen, Denmark) intraoral scanner, but this method is also suited for other powder-free intraoral scanners such as CEREC Primescan (Dentsply Sirona, Bensheim, Germany) or Medit i700 (Medit, Seoul, South Korea). The scanner has to be held at 90 degrees to the bur to minimize the level of noise, which can cause scanning errors [17]. The scanning protocol has to be performed following the instructions of the manufacturer: Start with the occlusal surface and keep the scanner 0–5 mm from the tooth. Roll the scanner’s tip 45°–90° to the buccal and lingual sides [18]. Due to the cylindrical shape of the bur, its scanning protocol is similar to that of implant scan bodies where the clinician has to make a circular movement around the scan body [19] (Figure 8).

However, an additional way to validate the precision of the reference plane is to use the color scale of the CAD software, which can identify differences down to 10 microns. This method is less accurate than scan body solutions provided by other companies, but they are more expensive and not always free of precision risks. Other studies are needed to verify this method.

The workflow described can be used to manufacture in-office hybrid posts and cores in a few hours. Therefore, the doctor can deliver the restoration to the patient on the same day, without the need for a dental laboratory. It is important to keep in mind that the clinician must have an in-office four-axis milling machine that can mill the zirconia core: CEREC MC XL (Dentsply Sirona, Bensheim, Germany), VHF Z4 (VHF Camfacture, Ammerbuch, Germany), or DWX-4 (Roland DG Corporation, Hamamatsu, Japan). The milling machine is used together with a high-speed sintering furnace that can sinter a zirconia core in less than an hour: CEREC SpeedFire (Dentsply Sirona, Bensheim, Germany) or LHT 01/16 Turbo Fire (Nabertherm, Lilienthal, Germany) [20]. The zirconia block used for this method is Katana (Kuraray Noritake, Tokyo, Japan), but the clinician can use blocks from any other manufacturer: IPS E.Max ZirCAD (Ivoclar Vivadent, Schaan, Liechtenstein) or InCoris (Dentsply Sirona, Bensheim, Germany).

Additionally, the design of the core is carried out using Exocad (Exocad GmbH, Darmstadt, Germany) version DentalCAD 3.0 Galway. However, this novel method can be used by any other CAD-CAM technology from different developers such as Dental System (3Shape, Copenhagen, Denmark) version Dental System 2022 or CEREC (Dentsply Sirona, Bensheim, Germany) version CEREC SW 5.2.

## 4. Conclusions

The present paper proposes an easy and straightforward method for producing in-house hybrid posts and cores without the use of conventional impression materials. Thus, the patient’s comfort is increased. Additionally, the patient can receive the restoration in the same day, so the number of visits to the office is reduced.

## Data Availability

Not applicable.
